# Impact of the recent Ebola epidemic with pandemic potential on the economies of Guinea, Liberia and Sierra Leone and other West African countries

**DOI:** 10.11604/pamj.2021.40.228.28391

**Published:** 2021-12-16

**Authors:** Mohamed Lamine Dramé, Paulo Ferrinho, Maria Rosário Oliveira Martins

**Affiliations:** 1 Health Policy and Health System Expert, International Consultant, Port Moresby, Guinea,; 2 Global Health and Tropical Medicine, Instituto de Higiene e Medicina Tropical, Universidade Nova de Lisboa, Lisboa, Portugal

**Keywords:** Ebola, pandemic, socio-economic impacts, West African countries

## Abstract

West Africa experienced its first Ebola epidemic in 2014. Its magnitude in terms of morbidity and mortality was greater than any other epidemic. It has particularly affected Guinea, Liberia and Sierra Leone. Its impact, beyond the high mortality, is also economic. The Ebola virus disease spread to several other African countries with limited resources, causing a significant financial burden to their health systems but also impacting the entire economy of the countries. The objective of this essay is to reflect on the consequences of the Ebola virus epidemics on West African economies in the short term. Estimates of the economic burden of the epidemic range from $2.8 billion to $32.6 billion in lost gross domestic product. The sectors affected by the economic crisis are the most important of the contaminated countries, namely agriculture, mining and trade. There has been a halt in socio-economic activities in the most affected regions. The decrease in the number of workers affected by the virus, the exodus to the least affected areas, and the repatriation of government employees have contributed to the decrease in the income of individuals and states. The fear of contamination by foreign countries has reduced imports, but also all tourist activities, which in turn have had an impact on the restaurant and hotel sectors. All these financial and food disruptions have exposed the population of these countries to food insecurity. The analysis of the impact of the Ebola virus on West African economies in the short term was as devastating as the health impact. This impact has directly contributed to a decrease in economic growth not only for the affected countries but also for all West African countries that depend on these same resources. A loss of about US$32.6 billion over two years in the West African region has been estimated, which is equivalent to 3.3% of the regional gross domestic product (GDP) in the absence of Ebola in 2014.

## Essay

Ebola virus disease, also known as Ebola haemorrhagic fever, is a serious, often fatal disease in humans. It first appeared in 1976 in the provinces of South Sudan and the Democratic Republic of Congo. A major outbreak of the Ebola virus occurred between 2014 and 2016 in West Africa. Three countries have been particularly affected by this outbreak: Guinea, Liberia and Sierra Leone [1]. This epidemic was more devastating than all other epidemics combined [2,3]. Unlike previous epidemics that were easier to contain, the West African epidemic has had a greater impact, particularly because of the time it lasted, the rapid spread, the unprecedented mortality and morbidity rates, and the real potential for global transmission [4]. The World Health Organization (WHO) reported a total of at least 28,000 cases and more than 11,000 deaths, as well as more than 10,000 survivors with after-effects [1,5]. However, this figure appears to be underestimated and should in fact be close to more than 20,000 deaths due to the difficult accessibility of certain areas to medical teams [4]. The Ebola virus disease spread to several African countries with limited resources, causing a significant financial burden to their health systems but also impacting the entire economy of the countries [6]. In this essay we reflect on the consequences of the Ebola virus on West African economies in the short term, supporting our arguments on some of the relevant literature available [7]. This essay facilitates the study of the economic weight generated by this outbreak on the three most affected countries: Guinea, Liberia and Sierra Leone as well as on other West African countries. This 2014 epidemic helps to explain in many angles the current economic performance of these countries and may help anticipate the magnitude of economic impact of the current COVID-19 pandemic in these countries.

**Economic burden of the epidemic:** estimates of the economic burden of the epidemic in West Africa range from $2.8 billion to $32.6 billion in lost gross domestic product. The overall economic and social burden of the 2014 Ebola epidemic has been estimated at $53.19 billion. The largest component, estimated at US$18.8 billion, was due to deaths from causes other than the Ebola virus [2]. In West Africa, particularly in Guinea, Liberia and Sierra Leone, the main people affected by the virus were those working in the agricultural and mining industries. These sectors employ a large part of the population in this sub-region of Africa. For example, in Sierra Leone agricultural workers represent about 66% of the population, in Liberia 50%, in Guinea 80%, while in Nigeria 70% [8]. Therefore, this essay will focus on the impact of the Ebola virus on trade, agriculture, mining and other sectors of the economy.

**Impact on trade:** however, an indirect economic consequence of the epidemic had an even greater impact on West African trade, namely the profound wave of panic it generated throughout the world [3]. Indeed, fear of infection led many governments and private companies to restrict the movement of people and goods, resulting in the slowing down or even stopping of trade and commercial transactions with the countries most directly affected by the virus [3,6]. This panic also contributed to excess mortality due to the collapse of the health system in the three countries and some neighboring countries [4]. This, together with the high mortality rate of the epidemic, had a direct impact on the availability of labour, reducing the production capacity of companies.

**Collapse of the agricultural sector and food shortages:** the Ebola epidemic had a strong economic impact on agriculture. The reduced mobility of farmers and other agricultural workers, but also the difficulty of getting products to harbours due to the quarantine zone, prevented affected countries from being able to produce and sell their goods [4,8,9]. The epidemic killed and drove out many farmers, leading to the abandonment of fields whose plantations turned into rotten food. Some larger farms faced significant labour shortages as a result of quarantine and the migration of many families [8,10]. The lack of farm labour, was aggravated by the necessary evacuation of some management staff and supervisors, particularly in palm oil companies in Guinea and rubber companies in Sierra Leone. According to the World Bank in 2014, coffee production halved (from 5,736 tonnes to 2,671 tonnes), cocoa production fell by a third (from 3,511 tonnes to 2,296 tonnes) and palm oil production by 75% in these countries [11]. This led to severe food shortages and pressure on food prices. Reduced production of food and cash crops in these countries affected export volumes, leading to lower profits for producers and sellers, as well as lower household incomes [4,8]. Some food exports were also heavily impacted, further exacerbating food shortages, as Thai and other foreign buyers stopped importing rice from Ebola-affected countries for fear that their transport ship personnel might be contaminated [8]. Declining export opportunities, once again, led to higher food and cash crop prices in these countries [8].

According to the World Food Programme and the Food and Agriculture Organization, disruptions in markets and in the processing and distribution chains of agri-food products exposed nearly one million people to food insecurity [10]. This contributed to increased malnutrition, especially among women and young children, many of whom were vulnerable or already affected by the disease [11]. According to the World Bank short- and medium-term estimates for the three West African countries, the mining sector accounted for about 17% of GDP and 56% of the US$559 million in total exports in 2013 [11]. The Ebola threat led major international firms to curtail their activities or freeze their projects (ArcelorMittal, China Union, Rio Tinto, Vale), depriving the three most affected states of significant fiscal resources [10]. In Sierra Leone, the mining industry accounted for 87% of the industry, or about 20% of the country's economy. The Ebola virus has had little effect on mining production as companies have redoubled their efforts to maintain minimum production. Nevertheless, several of these companies have had to operate with a reduced number of staff, particularly expatriate staff, which has led to disruptions [8]. In Guinea, the mining industry was less severely affected than in the other two countries as well, since the main mines were not located in areas affected by the virus [8].

Impact on other sectors of the economy: the Ebola virus has also had repercussions on the economy in other areas. Sectors such as transport, tourism, hotels, restaurants, education and the health system have been strongly affected by the epidemic. Large companies have reduced or even stopped their activities and repatriated qualified expatriates (experts), which has led to a significant reduction in economic productivity [3,4,8,10,11]. Due to restrictions on movement across borders and in affected major cities, transport workers have had a lower return due to the scarcity of passengers. In Liberia, petrol and diesel prices fell by 21 and 35 per cent, the limitation of taxis to 4 passengers increased the cost of domestic travel, and the cost of transporting goods was reduced with an increase in some places of 50 per cent [8,11]. International air travel was strongly affected by the epidemic. British, Emirates and other airlines banned flights to Sierra Leone and Liberia. The number of flights to Liberia decreased from 27 weekly flights in August to 6 in early September and from 31 to 6 in Sierra Leone [11]. This decrease in air traffic in the affected countries, added to the fear of contagion, and resulted in a reduction in the number of tourists and thus in the tourism activity income of the countries [11].

The decrease in tourist activities led to disruptions in the restaurant and hotel business. As a result of the ban on air travel, the evacuation of staff from foreign companies and the cancellation of conferences and seminars, hotel occupancy fell significantly. Six months after the official announcement of the outbreak, low hotel occupancy rates were recorded: 40% in Guinea (compared to 80% before the crisis), 30% in Liberia (compared to 70% before the crisis), 13% (compared to 60-80% before the crisis) in Sierra Leone [10-12]. The catering sector also suffered greatly during the epidemic, as it was one of the sectors where the number of jobs decreased the most following several layoffs [2].The health sector was also deeply affected. First of all, a high mortality rate among health workers was observed. Fear of infection led to a decrease in the number of people attending health centres for other diseases. Public health interventions such as vaccination, antenatal care, diagnosis and treatment of common diseases such as diarrhoea, malaria, pneumonia, HIV/AIDS, tuberculosis, as well as effective emergency case management dropped sharply. For malaria in 2014, the Ebola epidemic was estimated to have led to an increase in malaria cases of 45% in Guinea, 88% in Sierra Leone, and 140% in Liberia, with an additional 10,000 deaths [4,13]. In the Ebola affected districts in Guinea, health facilities were badly affected. In November 2014, 94 health centres (23%) closed due to desertion and/or deaths of health workers [14].

Lessons learnt: West Africa is constantly faced with various health challenges, yet the Ebola virus caused a particularly deadly epidemic. This was further aggravated by its syndemic nature that led to epidemics within the epidemic, with synergistic interaction of diseases and social conditions at the biological and population levels, leading to excessive mortality from other diseases, from lack of care, from malnutrition and increased poverty [15]. The impact of the virus on West African economies in the short term was as devastating as the health impact. The impacts on the strongest economic sectors of West African countries, agriculture, manufacturing and services, resulted in economic loss for the affected countries. Cross-border trade and economies were also disrupted [15]. These directly contributed to a decrease in economic growth not only for the affected countries but also for the West African region that depended on these same resources. A loss of about US$32.6 billion over two years in the West African region was estimated, which is equivalent to 3.3% of the regional GDP in the absence of Ebola in 2014 [11]. The interrelationship between economic crisis and previous pandemics has been evoked by economists, policy-makers, public health officials and other actors in the past. But economic crisis after a pandemic is felt most strongly under pre-existing stressful economic conditions reflected in underdeveloped economies excessively dependent of the primary sector and informal markets in burgeoning urban slums. Tackling 'emerging diseases' is thus as much about reconfiguring financial and economic systems as it is about local and global economic and health resources [16].

Studies of the macroeconomic impact in past pandemics have attempted to quantify the consequences in terms of lost output and growth, with little consensus. Medical variables related to the pandemic are crucial for the estimation of its economic effects, namely the morbidity rate, the number of work weeks lost, and the mortality rate (the percentage of those infected that die). Data on rates of absenteeism, effects on demand and trade effects, length of the pandemic and season of the year when it happens are some of the other variables important to conduct a comprehensive analysis of macroeconomic impact of the pandemic. We did not find any study modelling all these variables [17]. The interconnectivity of humans, animals and the social and abiotic environment is also highlighted by pandemics and it is most relevant in understanding and tackling any threats to food systems, agricultural production and other systems of economic livelihood, particularly in economic systems where animals play an important role for society and food security-providing, income, transport, fuel and clothing as well as food, as it is the case in West Africa. These highlight the relevance of a holistic understanding of and response to the pandemic [4,10,18,19]. Thus, future policy approaches to pandemics should enable adopt strategies to tackle both (endemic) diseases and their determinants, and pandemic-specific factors and outcomes that lead to the clustering of health and economic vulnerabilities and disparities over time. This more holistic approach would integrate conceptual frameworks such as “one health”, “health in all policies” and assumes a sustainable development (SDG) framework in the solutions adopted [19-22].
